# A Review on P300 in Obsessive-Compulsive Disorder

**DOI:** 10.3389/fpsyt.2021.751215

**Published:** 2021-11-23

**Authors:** Alberto Raggi, Giuseppe Lanza, Raffaele Ferri

**Affiliations:** ^1^Unit of Neurology, G.B. Morgagni – L. Pierantoni Hospital, Forlì, Italy; ^2^Department of Surgery and Medical-Surgical Specialties, University of Catania, Catania, Italy; ^3^Clinical Neurophysiology Research Unit, Oasi Research Institute - Istituto di Ricerca e Cura a Cattarere Scientifico (IRCCS), Troina, Italy

**Keywords:** cortical hyperarousal, information processing, obsessive-compulsive disorder, over-focused attention, p300, translational neuroscience

## Abstract

Neuropsychological studies indicate the presence of cognitive changes in patients with obsessive-compulsive disorder (OCD). Indeed, OCD may be included among the dysfunctions of the frontal lobes and their connections with the limbic system, associative cortex, and basal ganglia. P300 is a positive component of the human event-related potential (ERP); it is associated with processes of encoding, identification, and categorization constituting, as a whole, the superior cortical function of information processing. Thus, P300 explores several areas that are implicated in OCD pathophysiology. Our aim is to review all relevant studies on the P300 component of the human ERP in order to recognize any significant central nervous system (CNS) correlate of cognitive dysfunction in OCD. A PubMed-based literature search resulted in 35 articles assessing P300 in OCD and reporting neurophysiological correlates of response inhibition, cortical hyperarousal, and over-focused attention. A decreased P300 amplitude was reported in both adult and pediatric patients, with a trend toward normalization after pharmacological treatment. Source localization studies disclosed an association between P300 abnormalities and the functioning of brain regions involved in the pathophysiology of OCD. Moreover, studies converge on the evidence of neurophysiological dysfunction in the frontal areas with impairment of the normal inhibitory processes in OCD. At least some of these electrophysiological correlates might reflect the obsessive thoughts and compulsions that characterize this disorder. These findings may also support cognitive-behavioral therapy (CBT) approaches on over-focused attention and inflexibility of compulsive behaviors, which should be associated to pharmacological treatment in these patients.

## Introduction

Obsessive-compulsive disorder (OCD) affects approximately 2–3% of people at some point of their life (1). In the Diagnostic and Statistical Manual of Mental Disorders— Fifth Edition (2), OCD was moved from the group of anxiety disorders to a new category, named Obsessive-Compulsive and Related Disorders (1). Obsessive-compulsive disorder usually first appears during adolescence or in early adults, although treatment may not be sought until the middle age. The two sexes are equally affected. The onset of OCD is typically gradual and cannot be accurately dated, although, in some cases, it is triggered by a particular event in the patient's life. In most instances, OCD is engrafted into a personality in which rigidity and lack of adaptability are prominent. These traits are manifest in the individual's punctuality and in his/her dependability in the activities of everyday life. Additionally, there is always a prevailing undercurrent sense of insecurity (3–6). Clinically, the main symptoms of OCD are intrusive thoughts or images (obsessions), which increase anxiety, and repetitive and ritualistic actions (compulsions), which typically decrease anxiety (7, 8). In all of these obsessions and compulsions, patients suffer from a feeling of insufficiency in being unable to reject their troublesome thoughts (7, 8). This insight into the psychopathological experience and the struggle against it distinguish obsessions from delusions. Therefore, the majority of OCD patients are tense, irritable, and apprehensive. They may complain of anxiety attacks and become depressed with fatigue and lack of interest (7, 8). Education and pharmacotherapy with selective serotonin reuptake inhibitors (SSRIs) are first-line treatments, along with behavior therapy (7).

Neuropsychological studies indicate that patients with OCD may present some cognitive changes mainly involving executive functioning, information coding, organization strategy, set-shifting, motor and cognitive inhibition, visual-constructive and controlled fluency, verbal memory, and processing speed (9–11). Because of its neurobiological features, OCD may be included within the wide spectrum of dysfunctions of the frontal lobes and their connections with the limbic system, the associative cortex, and the basal ganglia which, in turn, influence the ability of abstract decisions needed to create more efficient and controlled behavioral judgment or action (12). In a previous study of patients who developed elements of obsessiveness and compulsive behavior after focal brain lesions, the authors found changes in the cingulate, frontal, and temporal cortices, as well as in the basal ganglia (13). On the other hand, the surgical disconnection of the orbito-frontal regions from limbic, thalamic, and striatal structures in severely affected patients improves symptoms of OCD (14). Moreover, neuroimaging studies in OCD demonstrate the involvement of the cortico-striato-thalamo-cortical loop, anterior cingulate cortex, prefrontal cortex, cerebellum, and hippocampus (15–18).

Based on this theoretical background, in this review we aimed to assess the utility of the P300 in the identification of any central nervous system (CNS) alteration, possibly correlated with cognitive dysfunction, in OCD patients. In particular, we focused on the electrophysiological correlates of some behaviors of individuals with OCD, such as the tendency of these patients to become aroused and exhibit strong defensive reactions to minimal stimulation (19, 20). Another aim was to assess the possible contribution of the P300 to the characterization of different OCD expressions, e.g., structural vs. functional brain abnormalities, within the clinical spectrum of a disease with a likely multifactorial basis.

As known, P300 is a positive component of the human event-related potential (ERP). It is associated with processes of encoding, identification, and categorization, that constitute, as a whole, the superior cortical function of information processing, and it is influenced by the effects of natural (i.e., circadian, ultradian, seasonal) and environmental state variables, such as fatigue (21–25). P300 is generated along a widely distributed network, rather than by a specific region, including the hippocampus and the medial temporal lobe, the temporo-parietal junction, and parietal areas, although the inferior and middle frontal, the orbito-frontal, and the cingulate cortices also contribute to it (26). For this reason, P300 involves several areas that seem to be implicated in the OCD pathophysiology (12–18).

P300 is most commonly elicited with an oddball paradigm in which a subject detects an occasional target stimulus within a regular train of standard stimuli (23, 25). Another paradigm to elicit P300 is the so-called “Go/No-Go” task, in which subjects are required to respond to one of the choices but must withhold a response to the other alternative (27). The test is passed only when the “Go” condition is met and the “No-Go” condition fails (25, 27). In psychophysiology, Go/No-Go tests are used to measure the participant's capacity to sustain attention and to control responses during the electroencephalographic (EEG) recording and the acquisition of ERP waves for each answer (25, 27, 28). For instance, a “Go/No-Go” test can require a participant to perform an action given certain stimuli (e.g., press a button: “Go”) and to inhibit the same action under a different set of stimuli (e.g., not press the same button: “No-Go”) (25, 27).

P300, also referred to as P3b, is better recorded with a maximum peak over the Pz scalp location (according to the EEG international 10–20 system) if the subject is actively engaged in the task of detecting the target (25, 29). Otherwise, a distractor tone (novelty) usually elicits a P3a wave, that has a frontal/central maximum amplitude distribution and frontal sources (30, 31). The P3b amplitude is a function of some psychological variables, such as attention, expectation of the event, and attribution to the event of a significance and complexity of the task (22, 23). Wave latency provides an indirect measure of the duration of the processes involved in stimulus discrimination, and ranges approximately from 300 ms (for simple dual tone discrimination tasks) to 750 ms (for much more complex processes) (22).

The P300 has already been applied in depicting, better than neuropsychological tests, even subtle cognitive deficits in some neuropsychiatric diseases, both in adults and children, such as sporadic amyotrophic lateral sclerosis without dementia, narcolepsy, obstructive sleep apnoea syndrome, and migraine (32–36). Evoked potentials occurring after stimulus presentation and preceding (e.g., P100, N100, P200, N200) or following (slow wave) the P300 wave are all components reflecting the time course of task-related neural information-processing (25, 37–40). In detail, N200 is elicited by both expected and ignored rare stimuli; it is followed by P300 when the subject is engaged in a particular stimulus of recognition (40). N200 seems to be an automatic process independent of control; it is similar to P300 in terms of sensitivity to attention and stimulus infrequency, and its latency correlates with reaction time (37). Finally, it is worth reminding that P300 is not seen in the raw EEG recording and can only be detected by averaging (41).

In summary, P300 seems to be a tool to investigate possible and different information processing changes in patients with OCD. The number of studies published on this topic deserves a timely re-examination of the available literature. The conclusion arising from this review may lead to further insights into the research agenda of OCD and, translationally, into clinical applications in other psychiatric and neuropsychiatric disorders, in terms of diagnostic work-up, follow-up assessment, and pharmacological and/or behavioral treatment index of response.

## Methods

This review included all relevant original articles published in peer-reviewed journals, indexed in the National Institutes of Health—National Library of Medicine (PubMed) literature search system, from database inception to July 2021. Search terms were “obsessive-compulsive disorder” and “P300.” The main inclusion criterion required that all the original research articles measured the P300 wave in humans; conversely, all the studies that did not explicitly report data concerning the evaluation of P300 in OCD were excluded. Non-English written articles, book chapters, monographs, commentaries, reviews, case studies, dissertations, abstracts, and letters to editor were also excluded, as well as any other article that did not fit the primary goal of the present review. Titles and abstracts of the retrieved studies were independently reviewed by two authors (G.L. and R.F.) based on the inclusion and exclusion criteria. Additional articles in the reference list of the papers identified by the search were also evaluated for inclusion in the review.

## Results

**Figure 1:**
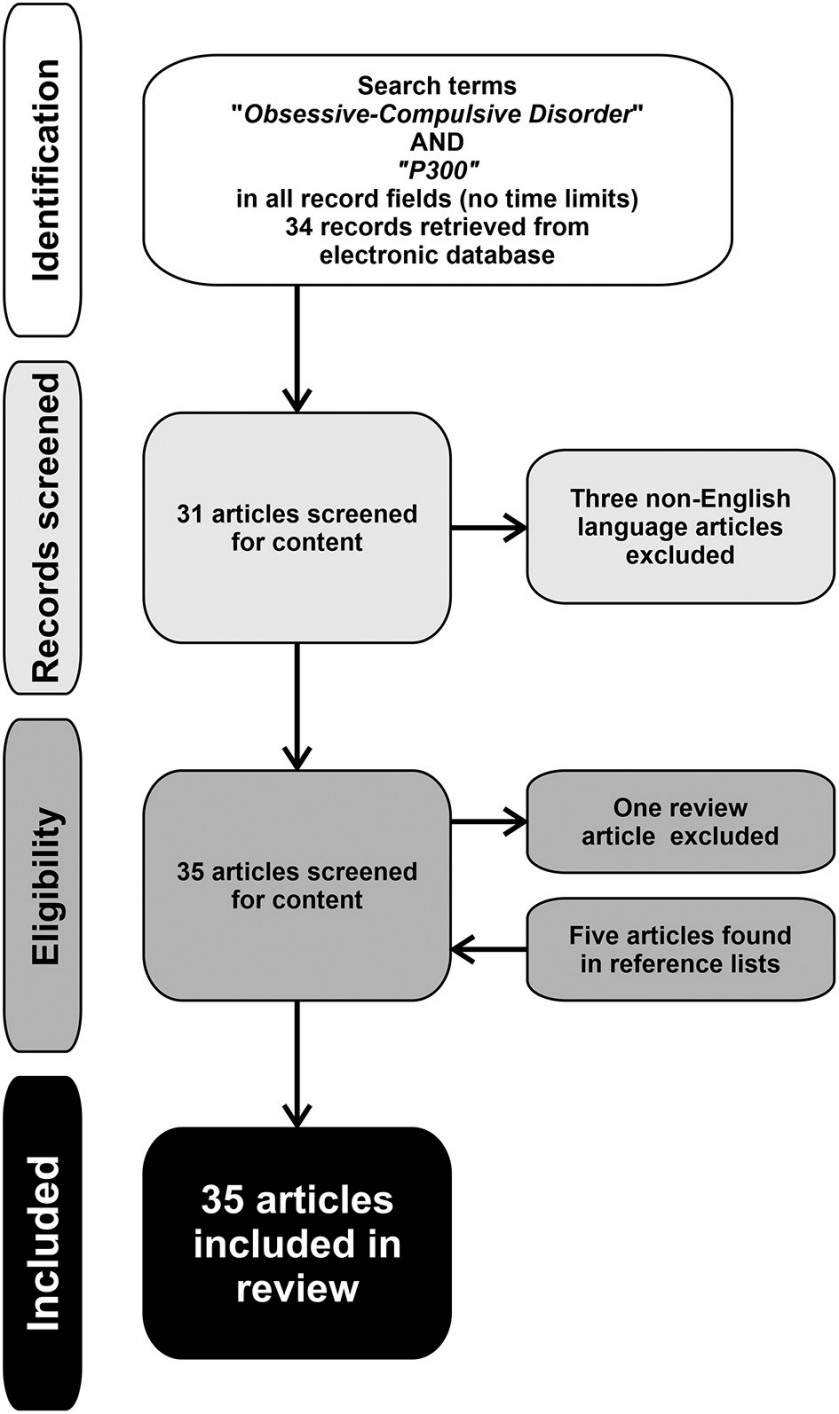
Flow of information through the different phases of the review process.

The PubMed-based search originally produced a group of 34 studies. Three of them were excluded because they were not written in English; another article was excluded because it was a review. Five additional papers were retrieved from the reference list of the selected articles. Therefore, a final group of 35 studies (summarized in Table 1) investigating P300 in patients with OCD was included in this review (19, 20, 28, 42–73). Figure 1 shows the flow of information through the different phases of the review process.

**Table 1:** Studies investigating P300 in patients with obsessive-compulsive disorder and P300 parameters at Pz (usually the lead with the largest peak).

**References**	**Groups (*n*)**	**OCD group age, years mean ± SD (range)**	**Study design, paradigm, main findings, and significance**	**P 300 amplitude**, **μV**		**P 300 latency, ms**	
				**OCD**	**Controls**	**OCD**	**Controls**
Ciesielski et al. (19)	8 unmedicated OCD (4 medicated), 8 HC	36.5, SD and range N/A	Cross sectional Visual oddball paradigm OCD patients showed P100 reduced amplitude and N200 decreased latency than the HC group; N.S. differences for the P300 OCD patients have a special potential for becoming aroused and exhibiting strong defensive reactions to minimal stimulation	N/A for Pz; 11.1 ± 4.9 (at P3 – DT); 10.8 ± 4.6 (at P4 – DT)	N/A for Pz; 16.0 ± 2.4 (at P3 – DT); 14.0 ± 2.8	N/A for Pz; 335 ± 23.5 (at P3 – DT); 333 ± 20.9 (at P4 – DT); (at P4 – DT)	N/A for Pz; 345 ± 10.5 (at P3 – DT); 349 ± 8.0 (at P4 – DT)
Beech et al. (20)	8 OCD (3 patients stopped antidepressant medication 48 h before testing), 8 HC	40, SD, and range N/A	Cross sectional Visual oddball paradigm OCD patients showed P300 reduced amplitude and decreased latency than the HC group OCD patients have a special potential for becoming aroused and exhibiting strong defensive reactions to minimal stimulation	N/A; 8.5 ± 2.5 (at P3 – ET); **7.1** **±** **2.8** (at P3 – DT); 8.6 ± 3.0 (at P4 – ET); **7.0** **±** **2.0** (at P4 – DT)	N/A; 10.8 ± 1.7 (at P3 – ET); **12.0** **±** **2.0** (at P3 – DT); 10.2 ± 2.1 (at P4 – ET); **12.4** **±** **2.9** (at P4 – DT)	N/A; **327** **±** **37.4** (at P3 – ET); **318** **±** **39.1** (at P3 – DT); **328** **±** **37.0** (at P4 – ET); **319** **±** **38.7** (at P4 – DT)	N/A; **356** **±** **21.0** (at P3 – ET); **365** **±** **26.5** (at P3 – DT); **357** **±** **27.4** (at P4 – ET); **368** **±** **23.1 7** (at P4 – DT)
Malloy et al. (28)	18 OCD (9 medicated), 18 HC	34 ± 12.8, range N/A	Cross sectional Go/No-Go visual test Topographic ERP mapping revealed significantly smaller P300 magnitudes in orbital frontal areas in the OCD patients Frontal dysfunction	26.2 ± 9.6 (Go); 25.9 ± 8.0 (No-Go); 10.9 ± 12.6 (Go at Fz); **14.3** **±** **12.8** (No-Go at Fz)	29.9 ± 13.07 (Go); 33.0 ± 12.5 (No-Go); 12.2 ± 14.9 (Go at Fz); **22.4** **±** **18.3** (No-Go at Fz)	N/A	N/A
Towey et al. (42)	10 unmedicated OCD, 10 HC	mean ± SD N/A (18–55)	Cross sectional Two-tone auditory oddball paradigm The OCD group showed significantly shorter P300 latencies and shorter N200 latencies for target stimuli with increasing task difficulty than the HC group; for both levels of task difficulty, OCD patients showed greater negativity than HC group in the N200 over the left hemisphere Cortical hyperarousal in OCD with a laterality pattern	N/A	N/A	N/A	N/A
Drake et al. (43)	20 unmedicated (10) or after a washout period of 1 week (10) GTS, 10 of whom had ADHD and 6 OCD	mean ± SD N/A (8–20)	Cross sectional Two-tone auditory oddball GTS patients with OCD had shorter N200 and P300 latencies Cortical hyperarousal in GTS patients with OCD as it happens in pure OCD patients	N/A	N/A	N/A; **272.0** **±** **41.3** (at Cz – GTS+OCD)	N/A; **358** **±** **13.7** (at Cz – GTS only)
Towey et al. (44)	17 unmedicated OCD, 16 HC	mean ± SD N/A (18–55)	Cross sectional Two-tone auditory oddball paradigm The OCD group showed significantly shorter P300 latencies and shorter N200 latencies for target stimuli with increasing task difficulty than the HC group; for both levels of task difficulty, OCD patients showed greater negativity than HC group in the N200 over the left hemisphere Cortical hyperarousal in OCD	N/A	N/A	408 (easy task); **402** (DT); SD N/A	395 (easy task); **456** (DT); SD N/A
Towey et al. (45)	17 unmedicated OCD, 16 HC	30 ± 9.1, range N/A	Cross sectional Two-tone auditory left and right presentation oddball paradigm The OCD group showed significantly larger attention-related PN than HC group; P300 amplitudes for attended targets were smaller for OCD patient than HCs, but the reverse was true for P300 for unattended non-targets Hyper activation of the frontal lobes	N/A	N/A	N/A	N/A
de Groot et al. (46)	18 unmedicated OCD, 18 HC	30.5 ± 6.9 (19–59)	Cross sectional Two-tone auditory oddball While not reaching significance, P300 latencies tended to be shorter for the OCD group; increased N200 negative amplitude and decreased latencies of the SW components; the more chronic the OCD symptoms, the more attenuated the integrated amplitude between 140 and 170 msec Cortical hyperarousal in OCD	N/A	N/A	325 ± 31	344 ± 39
Morault et al. (47)	13 unmedicated (1-week washout period with 5 responders and 8 non-responders) OCD, 13 HC	35 ± 8 (21–60)	Comparative study Verbal auditory oddball paradigm OCD patients showed longer latencies of the N100 and P200, shorter latency of the P300 and reduced amplitude of the N200; future responders to treatment had significantly reduced N200 and enhanced P300 amplitudes relative to future nonresponders. OCD patients stress the speed of task-dependent processes; ERPs might provide psychophysiological profiles in OCD patients with clinical and pharmacological implications	8.37 ± 4.29 (responders); 4.72 ± 6.28 (nonresponders)	N/A	**442.0** **±** **60**	**534.0** **±** **32.1**
Miyata et al. (48)	23 unmedicated OCD, 12 unmedicated SP, 18 HC	24.7 ± 5.0, range N/A	Cross sectional Two-tone auditory oddball paradigm The OCD group showed significantly shorter P300 latencies and shorter N200 latencies for target stimuli than the SP and the HC groups; there were no significant relationships between these ERP abnormalities in OCD patients and the type or severity of their OCD symptoms Shorter N200 and P300 latencies in OCD patients may be an OCD-associated phenomenon that is more closely related to the biological basis for OCD (cortical hyperarousal), rather than the characteristics of their OCD symptoms	13.5 ± 5.3	15.1 ± 6.1	**302.5** **±** **29.9**	**341.9** **±** **23.8**
Morault et al. (49)	21 unmedicated (1-week washout period) OCD, 21 HC	37.3 ± 10.9 (21–60)	Comparative and replication study Verbal auditory oddball paradigm OCD patients who were to respond favorably to treatment had significantly reduced N200 amplitude and shorter N200 and P300 latencies compared to non-responders and control subjects Some impairments of pre-treatment ERPs could be associated with future treatment outcome	N/A 3.5 ± 4.9 (G-mean) (responders); 1.9 ± 4.7 (non-responders)	N/A 2.2 ± 5.9 (G-mean)	N/A **466** **±** **72** (G-mean) (responders); 579 ± 6.0 (non-responders)	N/A **562** **±** **43** (G-mean)
Di Russo et al. (50)	8 unmedicated OCD, 12 HC	29.7 ± 6.3, range N/A	Cross sectional Discriminative response test OCD patients had greater P300 amplitude than HCs for the target stimuli, but not for non-target stimuli; spline map topography confirmed that P300 hyperactivation is localized principally on the frontal lobes Cortical hyperarousal in OCD as frontal lobe dysfunction	5.3 ± 0.7	N/A	N/A	N/A
Sanz et al. (51)	19 OCD, 19 HC	25.8, SD and range N/A	Cross sectional plus pharmacological follow-up study Two-tone auditory oddball paradigm P300 had lower baseline amplitude and longer latency in drug-free OCD patients when compared to HCs; P300 amplitude in OCD increased after treatment (clomipramine in 250-300 mg doses), although this was supported only by a statistical trend; there was no modification in P300 latency after treatment	**6.6** (SD N/A, drug free OCD)	**11.01** (SD N/A)	**308** (SD N/A)	**288** (SD N/A)
			The effect of treatment suggest that the cognitive function in OCD patients improved with pharmacological treatment possibly because of a better serotonin function, and this was reflected in a P300 amplitude close to that of normal people				
Mavrogiorgou et al. (52)	21 unmedicated OCD, 21 HC	33.9 ± 12.0 (17–57)	Cross sectional Two-tone auditory oddball paradigm OCD patients showed a larger P3b amplitude and a shorter P3b latency (only right hemisphere) as well as a shorter reaction time to target tones as the HCs The P3b abnormalities found in OCD patients could be an electrophysiological correlate of overfocussed attention and faster cognitive processes in OCD, possibly due to higher arousal	N/A	N/A	N/A	N/A
Herrmann et al. (53)	12 medicated OCD, 12 HC	41.2 ± 15.7, range N/A	Cross sectional Go/No-Go visual test Reduced frontal activity during the No-Go condition in OCD, which was condensed in a reduced anteriorization of the brain electrical field Frontal dysfunction	N/A	N/A	N/A	N/A
Kim et al. (54)	19 OCD (2 medicated), 22 SPR, 21 HC	26.74 ± 6.89, range N/A	Cross sectional Two-tone auditory oddball and NPS testing P300 amplitudes on all 15 electrode sites were significantly smaller in SPR and OCD patients than in HC subjects; P300 amplitude was related to the Trail Making Test (Part B) response time Frontal dysfunction	N/A	N/A	N/A	N/A
Kivircik et al. (55)	31 unmedicated OCD, 30 HC	27 ± 9.8 (18–55)	Cross sectional Two-tone auditory oddball paradigm The OCD group showed shorter P300 duration (calculated as the time difference between N200 peak and the beginning of the SW) compared to HCs; in NPS tests, no significant differences were found between the two groups Acceleration in the P300 process	7.40 ± 4.88	7.63 ± 4.33	N/A	N/A
Papageorgiou et al. (56)	18 OCD, 20 AHA, 20 HC	29 ± 10.3, range N/A	Comparative study Auditory working memory test AHA and OCD groups showed a reduction of the P300 amplitudes, located at the right frontal area as compared to HCs; the AHA exhibited a significantly lower P300 amplitude at central frontal areas relative to the other two groups; the OCD patients manifested a significant prolongation of P300 located at the central prefrontal area, relative to AHAs and HCs Both OCD and AHAs may share a common impairment of working memory and/or attention involving the right prefrontal areas.	14.9 ± 7.0	13.6 ± 5.8; 9.3 ± 5.5 (AHA)	331 ± 60; **326** **±** **67** (at Fz)	302 ± 57; 309 ± 71 (AHA); **275** **±** **73** (at Fz); **295** **±** **36** (AHA at Fz)
Kim et al. (57)	15 OCD (11 medicated, 4 unmedicated), 15 HC	25.73 ± 4.83, range N/A	Cross sectional Go/No-Go visual test The OCD patients manifested reduced No-Go-N200 and Go-N200 amplitudes at the frontocentral electrode sites compared to the HCs; the No-Go-N200 amplitudes and latencies measured at the central sites were also negatively correlated with the severity of symptoms; the OCD and HC groups were comparable with regard to Go-P300 and No-Go-P300 amplitude and latencies Dysfunctions in frontal regions mediating response inhibition in OCD detectable more by means of N200 than P300	10.76 ± 1.03 (Go); 5.86 ± 0.92 (No-Go)	8.72 ± 1.03 (Go); 6.67 ± 0.92 (No-Go)	413.00 ± 10.39 (Go); 415.87 ± 10.41 (No-Go)	419.13 ± 10.39 (Go); 410.60 ± 10.41 (No-Go)
Gohle et al. (58)	63 unmedicated OCD, 63 HC	33.71 ± 10.17, range N/A	Cross sectional Two-tone auditory oddball to elicit P300, which was separated with dipole source analysis into temporo-superior dipole (P3a) and temporo-basal dipole (P3b) OCD patients had significantly larger amplitudes of P3b than the HCs Study suggesting disturbances also in temporo-parietal and hippocampal regions in OCD	N/A 3.94 ± 2.3 (P3a); **7.05** **±** **2.42** (P3b)	N/A 3.75 ± 1.75 (P3a); **5.87** **±** **1.82** (P3b)	N/A 306.1 ± 25.8 (P3a); 320.6 ± 127.2 (P3b)	N/A 308.0 ± 23.7 (P3a); 316.8 ± 25.5 (P3b)
Thibault et al. (59)	15 medicated OCD, 14 GTS, 12 GTS+OCD, 14 HC	37 ± 13, range N/A	Cross sectional Visual counting oddball paradigm The P300 was reduced in participants in both OCD and GTS+OCD groups in the anterior region; the P300 oddball effect was significantly larger in participants of the GTS group compared to all other groups, mostly in the parietal region GTS is characterized by enhanced working memory updating processes and the superimposition of OCD could lead to a reduction of these processes	N/A	N/A	N/A	N/A
Pallanti et al. (60)	16 OCD, 11 schizo-OCD, 14 SPR, 12 HC	29.7 ± 6.3 (18–65)	Cross sectional Discriminative response test Schizo-OCD patients showed a distinct ERP pattern, with abnormally increased target activation (akin to OCD, but unlike the pattern observed in SPR) and reduced P300 amplitudes (akin to SPR, but unlike OCD); similar to HC; schizo-OCD patients showed larger amplitudes in the non-target condition than in the target condition Schizo-OCD may not only be a distinct clinical entity from pure OCD and SPR, but it may also be characterized by a distinguishable neurophysiologic pattern	6.30 ± 0.4	N/A	N/A	N/A
Ischebeck et al. (61)	20 OCD (10 medicated), 20 HC	32.8 ± 9.9, range N/A	Cross sectional Visual recognition test during which irrelevant repeated standard sounds and unitary novel sounds were interspersed Novelty P300 amplitude increased in OCD; scalp distribution of the novelty P300 was less lateralized in patients than in controls A physiological indicator of an enhanced cortical orienting response implicating stronger involuntary shifts of attention	N/A	N/A	N/A	N/A
Andreou et al. (62)	71 unmedicated OCD, 71 HC	34.68 ± 10.83 (18–62)	Cross sectional plus pharmacological follow-up study Two-tone auditory oddball with source localization analysis Increased P300-related activity was observed predominantly in the left orbitofrontal cortex, but also in left prefrontal, parietal and temporal areas, in patients compared to controls at baseline; after treatment, reduction of left middle frontal cortex hyperactivity was observed in patients Association between P300 abnormalities and activity in brain regions postulated to be involved in the pathophysiology of OCD	**7.43** **±** **2.89**	**6.47** **±** **2.63**	327 ± 52.2	340 ± 57.3
Endrass et al. (63)	25 OCD (8 medicated), 25 HC	33.4 ± 9.4, range N/A	Cross sectional A four-choice object reversal learning test measuring FRN and P300 Active task that required recurrent feedback-based behavioral adjustment in response to changing reward contingencies Higher error rates of OCD patients in response to negative feedback (FRN was reduced for negative feedback); the P300 was larger on all positive feedback events and on second exploration negative than on reversal negative feedback FRN reduction suggests attenuated monitoring of feedback during the learning process in OCD	N/A	N/A	N/A	N/A
Yamamuro et al. (64)	20 OCD, 20 HC	12.8 ± 2.5, range N/A	Cross sectional Two-tone auditory oddball paradigm The amplitudes of the P300 components in the Fz, Cz, Pz, C3, and C4 regions were significantly smaller in the OCD group compared to the HC group; there was significant correlation between illness severity and amplitude values at Cz, Pz, C3 P300 amplitudes are sensitive tools for measuring the biological aspects of OCD severity	**17.9** **±** **7.2**	**22.6** **±** **7.3**	315.5 ± 26.6	329.7 ± 17.8
Li et al. (65)	35 FC, 24 HC	mean ± SD N/A (18–70)	Observational study Go/No-Go visual test There was reduced P300 amplitude at F7 between FC and HC groups Cognitive dysfunction of implicit processing might be involved in the abnormality of visual communication and information processing	5.64 ± 3.74 (FC group)	4.75 ± 3.48	437.86 ± 127.84 (FC group)	477.09 ± 129.58
Ozcan et al. (66)	33 OCD, 18 sibling, 21 HC	35.3 ± 11.9, range N/A	Cross sectional Two-tone auditory oddball paradigm P300 amplitude was sorted as patients<siblings<controls; the logistic regression analysis showed that, higher P300 amplitude, better performance on block design test and faster completion of Stroop test would predict being in the control group, whereas higher P200 amplitude would predict being in the case (patient and sibling) groups Identification of potential NPS and ERP endophenotypes of OCD	**10.02** **±** **4.08**	**13.81** **±** **3.77**; **10.78** **±** **4.82** (siblings)	**315.37** **±** **26.67**	**334.88** **±** **30.4**; **312.77** **±** **29.04** (siblings)
Yamamuro et al. (67)	14 OCD, 10 HC	33.20 ± 9.84, range N/A	Longitudinal study of 1 year Two-tone auditory oddball paradigm OCD had decreased P300 amplitude at the baseline which was significantly increased at Fz, Cz, C3, and C4, indicating normalization, after 1 year of treatment P300 may be a useful tool for evaluating therapy in OCD patients	14.59 ± 1.25 at baseline; 17.34 ± 1.62 after 1 year	18.52 ± 1.37 at baseline	321.33.77 ± 7.61 at baseline; 332.77 ± 7.61 after 1 year	312.40 ± 10.93 at baseline
Yamamuro et al. (68)	12 OCD, 12 HC	13.50 ± 3.26, range N/A	Longitudinal study of 3 years Two-tone auditory oddball paradigm OCD had decreased P300 amplitude at Fz – Cz – Pz– C3 – C4, which increased partly at Fz and C4 in association with symptomatic improvements Utility of SSRIs in pediatric OCD and of ERPs for evaluating pharmacological effects in treatment-naïve pediatric OCD patients	**18.10** **±** **2.3**	**25.87** **±** **2.02**	332.92 ± 15.19	331.08 ± 18.76
Dayan-Riva et al. (69)	38 unmedicated OCD, 38 HC	23.82 ± 1.56, range N/A	Cross sectional Visual oddball paradigm OCD patients demonstrated significantly enhanced P300 amplitude over bilateral parietal areas in response to neutral stimuli; emotional valence reduced this effect such that OCD patients did not differ from HCs in P300 amplitude under the angry stimuli condition	N/A	N/A	N/A	N/A
			Results may represent distracted primary cognitive processes in OCD, possibly serving as a basic source for compulsion initiation				
Okazaki et al. (70)	15 unmedicated OCD, 15 HC	11.53 ± 2.90, range N/A	Cross sectional Two-tone auditory oddball paradigm P300 amplitude was significantly attenuated in the OCD group at Fz, C3, and C4, compared to HCs; OCD had altered reaction time P300 reduction as an index of brain dysfunction in OCD	16.30 ± 1.68; **7.95** **±** **0.85** (at Fz); **10.55** **±** **1.28** (at C3); **9.38** **±** **1.17** (at C4)	17.48 ± 1.18; **11.80** **±** **1.46** (at Fz); **14.78** **±** **1.47** (at C3); **14.36** **±** **1.57** (at C4)	319.07 ± 7.05; 327.08 ± 5.78 (at Fz); 322.27 ± 6.24 (at C3); 322.21 ± 5.47 (at C4)	327.20 ± 9.41; 324.53 ± 10.46 (at Fz); 327.33 ± 8.84 (at C3); 324.33 ± 9.64 (at C4)
Wojcik et al. (71)	30 psychiatric patients including 2 OCD	mean ± SD and range N/A	Containerization study Visual oddball paradigm with source localization analysis The most active Brodmann Areas for the few OCD patients were the frontal areas Hyper activation of the frontal lobes	N/A	N/A	N/A	N/A
Kloft et al. (72)	21 tic-free OCD (5 medicated), 12 tic-related OCD (5 medicated), 21 HC	34.0 ± 8.3 (tic-free OCD), 33.6 ± 8.8 (tic-related OCD), range N/A	Comparative study Visual/auditory stop-signal paradigm P300 amplitude was larger in tic-free compared to tic-related OCD and HCs Hyperactivity in the evaluation of the outcome of the inhibition process in OCD patients	N/A; **28.9** **±** **11.3** (at Cz – StC – tic-free OCD); **22.2** **±** **9.2** (at Cz – StC – tic-related OCD); **28.1** **±** **10.9** (at Cz – StF – tic-free OCD); **19.8** **±** **8.5** (at Cz – StF – tic-related OCD)	N/A; **22** **±** **6.7** (at Cz – StC); **19.4** **±** **8.4** (at Cz – StF)	N/A; 302 ± 32 (at Cz – StC – tic-free OCD); 326 ± 29. (at Cz – StC – tic-related OCD); 322 ± 21 (at Cz – StF – tic-free OCD); 340 ± 48 (at Cz – StF – tic-related OCD)	N/A; 306 ± 40 (at Cz – StC); 321 ± 24 (at Cz – StF)
Wolff et al. (73)	27 OCD (2 medicated, 11 with neuropsychiatric comorbidities), 27 HC	13.8 ± 2.34, range N/A	Cross sectional Go/No-Go visual test P300 amplitudes revealed a significant main effect of condition indicating significantly increased (more positive) P300 amplitudes during Go vs. No/Go-trials; a significant main effect of congruency was observed indicating significantly increased (more positive) P300 amplitudes during incongruent vs. congruent trials Pathological fronto-striatal hyperactivity and loss of a situation-specific modulation of response selection mechanisms in OCD	**32.4** **±** **2.24** (Go); **23.58** **±** **0.12** (No/Go); **29.11** **±** **2.18** (incongruent trials); **26.89** **±** **1.97** (congruent trials)	N/A	N/A	N/A

*ADHD, attention deficit hyperactivity disorder; AHA, abstinent heroin addicts; DT, difficult task; ERP, event-related potential; ET, easy task; FC, functional constipation; FRN, feedback-related negativity; G-mean, grand mean values across tasks and leads (Fz-Pz); GTS, Gilles de la Tourette syndrome; HC, healthy controls; N/A, not available; N.S., not significant; NPS, neuropsychological; OCD, obsessive-compulsive disorder; PN, processing negativity; SP, social phobia; SPR, schizophrenia; SSRI, serotonin reuptake inhibitor; StC, stop correct; StF, stop fail; SW, slow wave. Data are shown as mean ± standard deviation; statistically significant different values are in bold*.

Since the studies eventually included in this review deal with different aspects and methods, we will analyze their content in the following separate paragraphs.

### P300 Amplitude

Brain volume changes have been reported in OCD by neuroimaging (17). Therefore, we first consider here the studies reporting a decreased P300 amplitude, such as in neurodegenerative disorders (74, 75) or schizophrenia (76). In these conditions, a reduced amplitude of P300 is considered as a somewhat robust finding, possibly suggesting a genetic endophenotype (75, 76).

Overall, studies reporting a P300 amplitude reduction in OCD are thirteen (20, 28, 45, 51, 54, 55, 59, 64, 65, 67, 68, 70, 72), including the result of a P300 alteration at the F7 location in patients with functional constipation within a probable OCD (65). Conversely, we counted eight studies reporting enhanced P300 amplitude in OCD patients compared to normal controls (50, 52, 58, 61–63, 66, 69), a finding that was considered as an electrophysiological correlate of an OCD trait and indicating an increased propensity to be aroused (50, 77). Finally, one article reported that future responders to treatment had significantly enhanced P300 amplitude, compared to future non-responders; this finding is an example of the possible prognostic value of psychophysiological measures (47).

Clinical observations, neuropsychological testing, and pioneering neurophysiological investigations suggest deficits in set-shifting, impaired early-filtering selective attention, loss of normal inhibitory processes, and altered motor and cognitive inhibition in individuals with OCD (1, 7, 10, 11, 19, 20, 41). Accordingly, the “Go/No-Go” is a task in which subjects with impaired frontal lobe abilities are known to fail (78). Indeed, all the five studies adopting a “Go/No-Go” visual task in OCD highlighted a frontal dysfunction pattern (28, 53, 57, 65, 73), including a study in adolescent patients (73) and one in subjects with functional constipation and probable OCD (65). Therefore, the No-Go-N200 seems to be an accurate response inhibition measurement for patients affected by OCD.

A significant relationship between ERP abnormalities and severity of OCD symptoms was found in 13 studies (44, 46–53, 55, 57, 59, 64, 68–70, 72). Thus, ERP abnormalities might be considered as a sensitive tool for measuring the biological substrate of OCD severity. However, P300 characteristics were not associated with symptom severity in 11 studies on patients with OCD (19, 48, 51, 52, 54, 58, 61–63, 66, 67), thus allowing to hypothesize that P300 abnormalities in patients with OCD might constitute a trait, rather than a state, feature.

### P300 Latency

The shortening in P300 latency may reflect the trait of obsessionals to increase the response speed to task-dependent processes in a context of enhanced cortical responsiveness, probably due to a low level of inhibitory activity (20, 47). Studies reporting a decreased P300 latency in OCD are nine (20, 42–49, 52, 55). For instance, some authors using a two-tone auditory paradigm (55) described a shorter P300 duration in obsessional patients, compared to normal controls, calculated as the time difference between the N200 peak and the beginning of the slow wave. As stated, N200 is similar to P300 in terms of sensitivity to attention and stimulus infrequency, and its latency correlates with the reaction time (37). There are also several articles reporting N200 latency reduction (19, 42–44, 47, 48). Regarding the effect of treatment, one study only reported a longer P300 latency at baseline in drug-free OCD patients compared to controls, along with no modification at follow-up (51).

### Source Localization

A detailed knowledge of P300 generators is crucial for an appropriate understanding of its cognitive significance and clinical utility (79). However, this remains a challenging issue, especially when translating it to a practical level (e.g., the high number of EEG electrodes to be used) and when considering that a very large number of different generator assemblies can produce the same potential field on the scalp (80). As such, the localization of a limited number of equivalent dipoles is the most typical approach (81, 82) also used in OCD research.

A preliminary study was set up in order to perform a dipole source analysis for the discrimination between P3a and P3b subcomponents elicited by an auditory oddball paradigm. Obsessive-compulsive disorder patients showed a larger P3b amplitude and a shorter P3b latency, as well as a shorter reaction time to target tones, compared to healthy controls. The P3b abnormalities found in these patients might be viewed as the electrophysiological correlate of overfocussed attention and faster cognitive processes, possibly due to a higher arousal (52). However, it cannot be excluded that the abnormalities in P3b amplitude and latency may reflect structural or functional disturbances in temporo-parietal or temporo-basal areas of OCD patients. This view is supported by neuroimaging research showing hyperperfusion or activation in the medial temporal lobe of subjects with OCD (83). Larger amplitude and shorter latency of the P3a subcomponent, which mainly reflects the frontal hyperactivity (42, 45, 54), were not confirmed in this study, probably because the authors found no difference in the P3a between patients and controls. It is worth to note that this apparent negative finding may also shows some limits of the technique.

The orbito-frontal cortex, the activity of which was found to be impaired in OCD patients (17, 84), is located deeply in the ventromedial anterior part of the brain. Given that neuronal activity of the orbito-frontal cortex cannot probably be recorded by scalp electrodes, the temporo-superior dipole (P3a), which is calculated for the scalp data, was unable to reflect activity from this brain area. Subsequently, other authors confirmed the previous findings on the P3b (58), whereas a more recent study on 30 psychiatric patients (but only two with OCD) was carried out with a visual oddball paradigm followed by a source localization analysis using a 256-channel EEG dense array (71). The inferior frontal gyrus was found to be the most active brain area in the few OCD patients (71). Two additional studies (62, 69) involving a higher number of subjects supported the concept that P300 is generated along a widely distributed network involving several brain areas implicated in OCD, as recently confirmed by some neuroimaging research (17, 18), with both pharmacological treatment (62) and emotional drive (60) able to attenuate these functional changes.

Moreover, topographic ERP mapping revealed significantly smaller P300 magnitude in the rostral frontal areas during the “No-Go” condition in individuals with OCD (28, 53). In a subsequent study (57), patients manifested reduced No-Go-N200 and Go-N200 amplitudes at the frontocentral electrode sites, compared to healthy controls, although the two groups were comparable with regard to Go-P300 and No-Go-P300 amplitudes and latencies.

### Novelty P3a

The P3a originates from stimulus-driven frontal attention mechanisms during task processing (30). To obtain this evoked response, novel stimuli are presented infrequently within a background of frequently occurring standard stimuli and infrequently occurring distractor stimuli, while the subject is not required to respond mentally or physically to any stimulus (31). In an *ad hoc* study, novelty P3a amplitude was found to be increased in OCD patients compared to healthy controls, thus possibly representing a physiological index of enhanced cortical orienting response and implicating a facilitation of involuntary shifts of attention occurring in this condition (61).

### Laterality Pattern

A re-examination of positron emission tomography (PET), EEG, and single case studies previously performed by Flor-Henry (85) suggests that a lateralized dysregulation of the left fronto-caudate network is the major cerebral determinant of the obsessive-compulsive state. The concept is even more interesting when considering the recognition of features of OCD in patients with schizophrenia (86) and the hypothesized hemispheric imbalance in psychosis (87). The laterality pattern proposed by Flor-Henry (85) was partially confirmed by some N200/P300 studies and topographical mapping in OCD (20, 42, 44, 47, 49, 62, 65). Indeed, in a dipole source analysis, patients with OCD showed P300 impairment only in the right hemisphere (52). Moreover, both abstinent heroin addicts and individuals with OCD show a P300 amplitude reduction over the right frontal area, compared to healthy subjects (56).

### P300 and Treatment

We found six articles on the relationship between P300 and treatment in OCD (47, 49, 51, 62, 67, 68), one of them (68) was a pediatric survey. One study, and its replication with additional patients, showed that future responders to 1-year treatment (fluoxetine, fluvoxamine, clomipramine) had significantly reduced N200 and enhanced P300 amplitude compared to future non-responders (47, 49). Patients were considered non-responders if they failed to to respond to separate treatments lasting for at least 8 weeks in total, and at the maximum antidepressant dose for at least 5 weeks. The authors suggested that ERPs might constitute psychophysiological profiles in individuals with OCD, thus implying potential clinical and pharmacological implications (47, 49). Another article reported that P300 had a lower baseline amplitude and a longer latency in drug-free OCD patients compared to healthy controls; subsequently, P300 exhibited a trend toward an amplitude increase after treatment (clomipramine), without modification in latency (51). A few patients were treated when included in the study and initially recorded; they stopped taking medication, and after at least 1 month, when the acute symptomatology appeared again, they underwent the second interview and the ERP study. This allowed to minimize the learning effect on P300 obtained under treatment.

Some results by other authors (62) have already been described in the paragraph on source localization; the same authors also aimed to assess the effects of 10 ± 1 weeks of treatment with sertraline on P300 brain activity patterns. In the patients retested after treatment, a reduction of P300, in both amplitude and latency, was observed which, however, did not reach statistical significance, as also found by other researchers (51). On the contrary, another investigation demonstrated that individuals with OCD had decreased P300 amplitude at baseline, which significantly increased at Fz, Cz, C3, and C4, indicating normalization, after 1 year of behavioral and pharmacological treatment (fluvoxamine, paroxetine, sertraline, clomipramine); the same group achieved similar results on the utility of SSRIs in pediatric OCD patients (68). Compared to controls, P300 amplitudes were smaller in the OCD group at Fz, Cz, Pz, C3, and C4. Approximately 3 years after the start of SSRI treatment (unspecified molecules), P300 amplitude significantly increased at Fz and C4, along with clinical improvement.

### P300 in OCD Overlapping With Other Disorders

Since OCD can affect patients with other conditions, we also found some studies on OCD overlapping with other diseases, such as schizophrenia (60), Tourette syndrome (43, 59), functional constipation (65), mild depressive disorder, chronic motor or vocal tic disorder, social anxiety disorder of childhood, adjustment disorder, attention deficit-hyperactivity disorder (ADHD), social phobia, and expressive language disorder (73).

Comorbid schizophrenia-OCD (schizo-OCD) is characterized by the concurrent presentation of psychotic and obsessive-compulsive symptoms (86, 88–90), that may require high antipsychotic dosages for its acute exacerbations and for the maintenance of reduction of the severity of psychosis (91). Schizo-OCD sufferers show a distinct ERP pattern, with abnormally increased target activation similar to that described in OCD (20) but different from that usually observed in schizophrenia (76). These patients were reported to have also reduced P300 amplitude, similarly to schizophrenia (76, 92), but different from other results in individuals with OCD (50, 52, 58, 61–63, 66, 69). Therefore, schizo-OCD may be not only a clinical entity different from pure OCD and schizophrenia, but also a relatively distinct neurophysiologic condition (60).

Tourette syndrome is a neurodevelopmental disorder mainly characterized by tics, although most patients also experience sensory disturbances, especially in terms of premonitory urges and sensory hypersensitivity, which may account for comorbid OCD, ADHD, and autism spectrum disorder, with a possible partially common pathophysiology underlying them (93). One study described that patients with Tourette syndrome and OCD had shorter N200 and P300 latencies (43), thus confirming the above-mentioned common cortical hyperarousal state hypothesized for both conditions (93). Another article seems to be also in line with neuroimaging findings (18, 94) when describing a P300 amplitude reduction in the anterior scalp regions in both OCD and Tourette syndrome with OCD patients (59).

A study in patients with functional constipation found that they were also obsessive, anxious, and depressed, with reduced P300 amplitude at F7 compared to controls (65). The authors speculate that, in patients with functional constipation, asymmetric forebrain abnormal activities in the two hemispheres might initiate some implicit automatic processing, such as somatization and OCD, in order to cope with painful experiences caused by anxiety and depression.

Cognitive dysfunction of implicit processing might also be involved in impaired visual communication and information processing. One of the above-listed studies (73) did not consider comorbidities separately due to the low number of overlapping psychiatric conditions. The most active brain regions in the few OCD patients included were the frontal areas.

### P300 in OCD Compared to Other Conditions

Shorter N200 and P300 latencies in OCD compared to social phobia and normal controls were believed to be an OCD-associated phenomenon (speeding of cognitive processing) (48). In two studies, P300 amplitudes were significantly smaller in schizophrenia and OCD patients than in healthy subjects (54, 60), a finding which is in line with the hypothesis of brain volume changes in both psychiatric illnesses (18, 76, 92).

One group of researchers assessed working memory and attentional capacities in OCD and opioid addicted: the abstinent group showed a notable delay of the P300 latency compared to controls and OCD only over the right occipital region, while OCD patients exhibited a significant prolongation of the P300 recorded over the central prefrontal area compared to addicts and healthy controls (56). Although this was a rather complex study, the results of which were not fully in line with previous data on the P300 latency in OCD, it focused on the peculiar phenomenological aspect that addicts are quite similar to obsessionals when craving for drugs becomes irresistible, such as an obsession (95, 96).

P300 was reduced in participants with both OCD and Tourette syndrome and OCD over the anterior scalp regions, whereas the P300 oddball effect was significantly larger in participants with Tourette syndrome compared to all other groups (59). Therefore, the authors speculated that Tourette syndrome may be characterized by an enhanced working memory that updates processes and the superimposition of OCD might lead to a reduction of these processes (59). In one study only, P300 amplitude was found to be smaller in patients than in their siblings and also smaller in siblings than in controls; a logistic regression analysis showed that higher P300 amplitude and better performance at neuropsychological tests of the frontal cortex function were predictors for control subjects, whereas higher P200 amplitude predicted both patients and their siblings (66). The authors concluded that this pattern might be an endophenotype of OCD.

### Pediatric Studies

We found five articles on P300 in pediatric OCD patients (43, 64, 68, 70, 73). Some investigators emphasized that pediatric patients have reduced P300 amplitude (64, 68, 70), altered response time (70), and partial increase of P300 amplitude after SSRI treatment (68). The replicated finding of decreased P300 amplitude in OCD children and adolescents and the correlation between illness severity and P300 amplitude led the authors to suggest that this psychophysiological feature might be considered as a sensitive tool for measuring the biological aspects of OCD severity (64, 68).

Another study investigated the auditory information processing in children and adolescents with Tourette syndrome overlapping with ADHD or OCD (43). Tourette syndrome patients with OCD had shorter N200 and P300 latencies, indicating cortical hyperarousal, similarly to pure OCD patients (20, 42, 44, 47–49, 52, 55). This pivotal concept, i.e., the fact that OCD patients may have a distinctive tendency to be aroused and to exhibit strong defensive reactions to minimal stimulation, as already highlighted by some authors (19, 20) for adults with OCD, seems to be present also in young patients. Pediatric OCD patients showed higher P300 amplitudes during “Go” vs. “No/Go” trials and during incongruent vs. congruent trials, thus confirming abnormal frontal hyperactivity also in young OCD patients (73).

## Discussion

This review included 35 studies with different paradigms (e.g., classical visual or auditory oddball, novelty, Go/No-Go), which have different recording modes (from only a few to 256 electrodes) and several objectives (e.g., localization of dysfunctional brain areas and treatment response) in adults, adolescents, and children. This heterogeneity did not allow us to perform any meta-analytic calculation, although the most relevant findings about P300 in OCD have been addressed in this review and summarized in Table 2.

**Table 2:** Summary of the relevant data found in obsessive-compulsive disorder regarding the event-related potential components considered in this review.

**Feature**	**Finding**	**Main translational implication**
P300 amplitude	↑ or ↓	Possible different expressions (structural vs. functional brain abnormalities) within the OCD clinical spectrum
P300 latency	↓	Cortical hyperarousal
N200 latency	↓	Cortical hyperarousal with overfocused attention
Sources	Frontal and temporo-basal areas	Support neuroimaging findings of the involvement of cortico-striato-thalamo-cortical loop, anterior cingulate cortex, prefrontal cortex, and temporal areas
Novelty (P3a amplitude)	↑	Enhanced cortical orienting response implicating stronger involuntary shifts of attention
No-Go-N200 amplitude	↓	Frontal dysfunction pattern
ERP Laterality	R<L more frequently R>L less frequently N/A for many studies	The hypothesis of a lateralized dysregulation of the left fronto-caudate network is only partially supported by this data re-examination
Effects of SSRI on P300 amplitude	↑	Partial improvement that seems to be mainly attributable to the effect of serotonin on ERPs
Effects of behavioral therapy on P300	N/A	Necessity for this investigation in future research agenda
Overlap syndromes	ADHD, FC, SPR	Shared patterns of frontal damage or dysfunction has been related to other psychiatric disorders in addition to OCD
Matching conditions	AHA, GTS, SP, SPR	Frontal dysfunctional patterns similar to that of OCD for SP and somewhat different regarding the other disorders
Symptom severity and P300 amplitude	↑ or ↓	ERP abnormalities as a sensitive tool for OCD biological feature of frontal damage vs. ERP abnormalities as per OCD dysfunctional trait
Pediatric studies (P300 amplitude)	↓	Suggesting frontal damage
Pediatric studies (P300 latency)	= or ↓	Not relevant vs. speeding of cognitive processing

*ADHD, attention deficit and hyperactivity disorder; AHA, abstinent heroin addicts; ERP, event-related potential; L, left; N/A, not applicable; FC, functional constipation; GTS, Gilles de la Tourette syndrome; OCD, obsessive-compulsive disorder; R, right; SP, social phobia; SPR, schizophrenia; ↑, increased; ↓, decreased; =, unchanged*.

Neuroimaging-based multimodal approaches have led to the conclusion that subtle brain structural changes and functional abnormality are present in OCD (15, 17, 18), with PET studies showing hypermetabolism in the orbital frontal cortex of these patients (85, 97). In the context of structural changes in OCD, the value of a decreased amplitude of P300 becomes evident, as reported by several studies (28, 45, 51, 54, 55, 64–68, 70, 72), similarly to those performed in schizophrenia (77). The finding of attenuated P300 amplitude has also been considered to be a pattern of genetic endophenotype in OCD (75, 76), which has also been confirmed in pediatric studies (64, 68, 70). Moreover, it was found to correlate with illness severity, suggesting that the reduction in P300 amplitude may be a sensitive tool for measuring some biological aspects of OCD severity (64, 68), also based on the evidence that P300 is generated along a widely distributed network that includes several brain areas implicated in the pathophysiology of OCD (12–18, 26). Of note, one study in schizo-OCD patients showed a pattern of alteration, i.e., a reduced P300 amplitude as in schizophrenia (60), which suggests the occurrence of brain structural abnormalities also in this peculiar clinical psychosis with relevant obsessional symptoms (88–90).

Nevertheless, there are also studies, depending on the type of paradigm used but suggesting the possible existence of different expressions of OCD within its clinical spectrum, reporting enhanced P300 amplitude in patients with OCD, compared to healthy controls (50, 52, 58, 61–63, 66, 69). Most of these studies considered this result as the electrophysiological correlate of an OCD trait consisting in a special tendency to get aroused (50, 77). Notably, only one of these studies (69) revealed that there was a weak relationship between ERP abnormalities and symptoms severity in OCD; more specifically, the authors reported only a weak correlation between compulsion estimation and the P300 valence effect (69). Based on the enhanced P300 amplitude, a pattern of cortical hyperarousal and over-focused attention in OCD has been hypothesized (19, 20, 42–44, 46–50), as also supported by the findings on processing negativity, which were interpreted as an indication of the existence of a hyperactivation of the frontal cortical mechanisms (12, 16, 19, 20, 85).

The decreased N200 latency in OCD (19, 42–44, 47, 48) and P300 (20, 42–44, 47–49, 52, 55) further emphasizes the concept of dysfunctional speed of information processing, possibly leading to some clinical features, such as intrusive thoughts that increase anxiety. The loss of normal inhibitory processes (28, 53, 57, 65, 73) would then serve as a basic source for the initiation of compulsion, with the to decrease anxiety.

The pattern of different amplitude seems to suggest the presence of different expressions (structural abnormalities vs. brain dysfunction) within the clinical spectrum of OCD. For instance, patients with a severe symptomatology can be seen (64, 68), including those with schizo-OCD (60), as belonging to the first group (structural abnormalities), whose abnormalities mainly concern the frontal brain areas (85, 97). The dysfunction of the frontal lobes has been implicated in OCD (12, 85). Flor-Henry suggested that OCD may be secondary to a prominent frontal lobe dysfunction, along with a loss of the physiological inhibitory processes (98). Other studies confirmed the involvement of the cortico-striato-thalamo-cortical loop, the anterior cingulate cortex, and the prefrontal cortex in OCD (9–18, 85, 97). However, frontal damage or dysfunction has been found in a number of psychiatric disorders in addition to OCD, and particularly schizophrenia. Some neuroimaging studies indicate that OCD symptoms are associated with altered activity in the orbito-frontal cortex (12, 99, 100), being this finding likely due to the dominant role of the frontal lobe in executive functioning and self-regulatory behaviors (12, 100, 101), which are both altered in several psychiatric illnesses. Therefore, the frontal abnormality may reflect a final common pathway for abnormal behavior (12, 28). Alternatively, different disorders may result from the dysfunction of different frontal subsystems (12, 28). For instance, the dorsolateral frontal region has been found to be impaired in schizophrenia, whereas the orbito-frontal areas have been implicated in OCD (12, 102).

Nevertheless, the above-mentioned features may not fully explain the inconsistency between the enhanced and reduced P300 in OCD, thus making the interpretation of these results challenging. Furthrmore, the fact that several data have been published by the same research group needs be taken into account (64, 67, 68, 70). However, it should be noted that most OCD individuals show a peculiar propensity to get aroused and typically exhibit strong defensive reactions even to minimal stimulation (19, 20, 42–46, 48, 50, 55, 71, 72). Translationally, these are the patients who can benefit from a targeted cognitive-behavioral rehabilitation, associated with drug therapy. On the contrary, pediatric subjects, schizo-OCD patients, and severe OCD cases frequently show low-amplitude P300 (28, 45, 51, 54, 55, 64, 65, 67, 68, 70, 72), a finding in line with the brain imaging data reported by different authors (15–18). Based on the P300 parameters, follow-up studies after drug and rehabilitation therapy are warranted in order to gain further insights on the disease severity and the possibility of clinical and cognitive improvement (64, 103). In this scenario, it is worth to highlight that Morault et al. (49) have already suggested that some pre-treatment features of ERPs might be associated with a more favorable outcome after treatment.

The laterality pattern, i.e., left<right, proposed by Flor-Henry (85), was partially confirmed by some N200/P300 studies and topographic mapping in OCD (20, 42, 44, 47, 49, 62, 65), whereas the partial recovery of P300 changes, in parallel with the clinical improvement (47, 49, 51, 62, 67, 68), seems to be mainly attributable to the effect of serotonin on ERP amplitude (68).

Regarding the effect of treatment, only one study (67) used psychotherapy combined with drugs. In this context, two studies have provided useful psychophysiological profiles in individuals with OCD with clinical and pharmacological implications, thus suggesting that the outcome of cognitive-behavioral therapy (CBT) combined to SSRI should be studied along with ERPs (47, 49). In addition, there were also a few reports of P300 amplitude with partial recovery after adequate pharmacological treatment (51, 67, 68, 70).

## Conclusions and Future Directions

Notwithstanding the limitations of the studies and their heterogeneous methodology, the findings reviewed here seem to support that the different P300 patterns observed might suggest the presence of different expressions (structural vs. functional brain abnormalities) within the clinical spectrum of OCD. Event-related potentials may also be used as a treatment monitoring marker at the individual level, especially for both pharmacological treatment and/or CBT (104). In particular, the development of novel P3a paradigms in combination with P3b tasks seems to be promising for a more extensive application and reliability of ERPs (103). Moreover, similarly to patients with migraine, who can be re-tested during treatment and follow-up in order to detect an improvement of P300 habituation (35), also OCD patients might be serially evaluated over time to identify any possible change in their neurophysiological correlates of cortical hyperarousal, over-focused attention, and response inhibition. The same holds true for children and adolescents with decreased P300 amplitude at baseline, who might be re-evaluated during or after treatment in order to assess whether P300 changes reflect pharmacological and/or psychotherapeutic effects (67, 68).

Nevertheless, the majority of the articles reviewed here included very small cohorts of patients, thus emphasizing again the importance of objective evaluations in neuropsychiatric and psychological disorders, as well as the need for a systematic examination and the application of standardized procedures to obtain more realiable guidelines in the near future. For instance, it would be intriguing to apply the three-oddball paradigm (31) to record N100, MMN, and P3a with the same conscruct. Their abnormality would suggest a frontal dysfunction and might help the differentiation between OCD individuals, severe cases, and schizo-OCD. Other studies may deal with the lack of P300 habituation by using two or three blocks of stimuli, as already done in patients with migraine (35, 105). Finally, further data using multidimensional measurement techniques (e.g., behavioral, electrophysiological, structural, metabolic) will be also necessary before the relationship between brain (mainly frontal) dysfunction and OCD psychopathology can be conclusively clarified.

## Author Contributions

AR, GL, and RF designed research, performed literature review, analyzed data, wrote the paper, and revised the text. All authors contributed to this manuscript and agree with its content.

## Conflict of Interest

The authors declare that the research was conducted in the absence of any commercial or financial relationships that could be construed as a potential conflict of interest.

## Publisher's Note

All claims expressed in this article are solely those of the authors and do not necessarily represent those of their affiliated organizations, or those of the publisher, the editors and the reviewers. Any product that may be evaluated in this article, or claim that may be made by its manufacturer, is not guaranteed or endorsed by the publisher.
